# Explainable Machine Learning for Predicting Major Adverse Renal and Cardiovascular Events in Acute Myocardial Infarction With Chronic Kidney Disease: Algorithm Development and Validation

**DOI:** 10.2196/96654

**Published:** 2026-10-23

**Authors:** Wang Feng, Ruihao Jiang, Wendi Huang, Chuanpeng Wang, Zhuoquan Wang, Jingwen Chang, Jinguo Cheng, Nianshao Chen, Xi Liu

**Affiliations:** 1 Wenzhou TCM Hospital of Zhejiang Chinese Medical University, Wenzhou, Zhejiang, China; 2 First Affiliated Hospital of Wenzhou Medical University, Wenzhou, Zhejiang, China; 3 Second Affiliated Hospital & Yuying Children's Hospital of Wenzhou Medical University, Xueyuan West Road, Lucheng District, Wenzhou, Zhejiang, 325000, China, 86 18857851776

**Keywords:** acute myocardial infarction, chronic kidney disease, nomogram, machine learning, major adverse renal and cardiovascular events

## Abstract

**Background:**

Major adverse renal and cardiovascular events (MARCE) have been proposed as a comprehensive end point reflecting both cardiac and renal outcomes in patients with acute myocardial infarction (AMI) and chronic kidney disease (CKD). However, existing prediction tools lack accuracy and generalizability for this high-risk population.

**Objective:**

This study aimed to develop and externally validate explainable machine learning models and a nomogram for predicting in-hospital MARCE among patients with AMI and CKD.

**Methods:**

We enrolled patients with AMI and CKD who underwent emergency treatment at the First Affiliated Hospital of Wenzhou Medical University as the discovery cohort and those at Wenzhou TCM (traditional Chinese medicine) Hospital of Zhejiang Chinese Medical University as the external validation cohort. Clinical features, including demographic details, comorbidities, laboratory results, echocardiography measurements, procedural details, and medication parameters, were collected for all participants. Important variables were selected using least absolute shrinkage and selection operator regression in the training set. We developed extreme gradient boosting (XGBoost), random forest, decision tree, and logistic regression (LR) models and evaluated their discrimination, calibration, and classification metrics in the hold-out test set and external cohort.

**Results:**

In the training set, we identified 6 important variables (Killip class, lactate, estimated glomerular filtration rate, use of diuretics, use of beta-blockers, and use of renin-angiotensin system inhibitors). In the test set, model discrimination was comparable across XGBoost (area under the receiver operating characteristic curve [AUC] 0.882, 95% CI 0.816-0.947), LR (AUC 0.875, 95% CI 0.799-0.950), and random forest (AUC 0.870, 95% CI 0.800-0.940), while decision tree showed lower performance (AUC 0.814, 95% CI 0.720-0.886). Shapley additive explanations (SHAP) values illustrated both global and individual feature contributions in the XGBoost model. A nomogram based on LR achieved an AUC of 0.873 (95% CI 0.714-0.926) in external validation.

**Conclusions:**

Our study identified 6 important predictors and provided a series of prediction models to enhance risk stratification of MARCE for patients with AMI and CKD. These models might aid in stratifying risk and choosing appropriate therapeutic strategies in the management of patients with AMI and CKD.

## Introduction

Acute myocardial infarction (AMI) represents a major global public health challenge associated with significant mortality and disability. It is characterized by acute myocardial ischemia resulting from complete or incomplete occlusive thrombosis secondary to rupture or erosion of coronary atherosclerotic plaques [1]. Chronic kidney disease (CKD) is a strong independent risk factor for cardiovascular disease [2], affecting up to 30% to 40% of patients with AMI [3]. Compared with patients without CKD, those with AMI and comorbid CKD exhibit 2 to 5 times higher in-hospital mortality [4]. Reported rates of in-hospital major adverse cardiovascular events and acute kidney injury requiring dialysis range from 7.1% to 32.5% and 0.8% to 11.5%, respectively [5,6]. These poor outcomes are primarily attributed to frequent ineligibility for sustained reperfusion therapy and evidence-based pharmacotherapy, chronic volume overload, high comorbidity burden, and delayed medical attention due to atypical symptoms [3,5]. Consequently, patients with AMI and CKD impose a heavy socioeconomic burden and represent a critical population urgently requiring improved prognostic outcomes in cardiorenal management.

Recognizing the close cardiorenal interplay, the American Heart Association (AHA) has endorsed major adverse renal and cardiovascular events (MARCE), encompassing both cardiovascular and renal adverse events, as a novel composite clinical end point for evaluating cardiorenal disease prognosis [7]. This clinically significant end point is considered to provide a more comprehensive assessment of overall patient risk. Nevertheless, widely used traditional clinical prediction tools such as the Global Registry of Acute Coronary Events (GRACE) score and Cleveland Clinic score [8,9] exhibit critical limitations: they predominantly focus on single-organ end points (either cardiac or renal events) and fail to integrate the complexity of MARCE in cardiorenal comorbidity. More importantly, as approximately 75% of coronary heart disease-related clinical trials exclude patients with CKD [10,11], the applicability and predictive performance of existing models developed in “relatively healthier” populations are severely compromised when applied to real-world patients with AMI and prevalent CKD. Thus, there is an urgent clinical need for a robust novel tool to accurately predict MARCE risk in this high-risk population.

Machine learning (ML) methods demonstrate significant advantages in capturing complex nonlinear relationships and higher-order interactions within multidimensional data [12]. They have been extensively applied across medical research domains, including risk stratification and prognostic prediction [13,14]. Previous studies have demonstrated that ML-based models outperform traditional scoring systems in predicting adverse cardiovascular outcomes in patients with AMI [15,16]. However, widespread adoption of ML models in clinical decision support faces a major challenge: limited interpretability due to their inherent “black-box” nature [17,18], which hinders clinicians’ understanding and trust in model predictions. To address this limitation, this study integrates advanced ML algorithms with a Shapley additive explanations (SHAP)–based explainable AI framework. SHAP provides intuitive, individualized feature contribution explanations.

In this study, we aimed to develop and externally validate ML models for predicting in-hospital MARCE risk in patients with AMI and CKD. The models were designed to assist clinicians in identifying individual risk drivers, assessing disease severity, and supporting early risk stratification during index hospitalization.

## Methods

### Study Design

Based on predefined inclusion and exclusion criteria, this retrospective cohort study enrolled patients with AMI and CKD who presented and underwent emergency treatment at the First Affiliated Hospital of Wenzhou Medical University between January 2013 and July 2019. This group was designated the “discovery cohort” for model development and internal validation. An external validation cohort consisted of patients meeting the same criteria admitted between January 2020 and August 2021 at Wenzhou TCM (traditional Chinese medicine) Hospital of Zhejiang Chinese Medical University.

Inclusion criteria were as follows: (1) age 18 years or older; (2) diagnosis of AMI according to established guidelines [1]; (3) diagnosis of CKD, defined as an estimated glomerular filtration rate (eGFR) of <60 mL/min/1.73 m^2^, calculated using the Chronic Kidney Disease Epidemiology Collaboration (CKD-EPI) equation [19] from the first serum creatinine measured within 24 hours after admission; and (4) receipt of emergency treatment. Exclusion criteria included (1) death during intervention or within 24 hours of admission; (2) unavailability of coronary angiography images; (3) current dialysis or renal replacement therapy before admission; and (4) missing data exceeding 20% of individual patient records.

### Ethical Considerations

The study protocol received approval from the ethics review boards of both the First Affiliated Hospital of Wenzhou Medical University (number R2152025) and Wenzhou TCM Hospital of Zhejiang Chinese Medical University (number WZY2024-KT-0294‐01) and conformed to the Declaration of Helsinki. The retrospective design exempted the requirement for participant consent. This study was reported in accordance with the TRIPOD (Transparent Reporting of a Multivariable Prediction Model for Individual Prognosis or Diagnosis) statement (Checklist 1) [20].

### Data Collection

All data were extracted from the electronic medical record system within the first 24 hours of admission. The collected information included demographic characteristics, clinical presentation on admission, medical history, laboratory findings, echocardiography measurements, procedural details, and medication use.

Medical history, including hypertension, diabetes mellitus, and stroke, was identified based on *International Statistical Classification of Diseases, Tenth Revision* (*ICD-10*) codes. Laboratory results were obtained from tests conducted on the first day after admission. Echocardiography measurements were performed at admission, with left ventricular ejection fraction calculated using the modified Simpson biplane method from apical four-chamber and two-chamber views. Angiographic and procedural documentation included the degree and location of coronary stenosis, as well as the presence of calcification, thrombus, and whether thrombus aspiration was performed.

Medications administered during hospitalization, including beta-blockers, angiotensin-converting enzyme inhibitors (ACEIs)/angiotensin II receptor blockers (ARBs)/angiotensin receptor-neprilysin inhibitors (ARNIs), and diuretics, were also recorded.

Killip classification was defined as follows: class I indicated no signs of heart failure; class II was defined by the presence of rales and/or jugular venous distension; class III represented acute pulmonary edema; and class IV denoted cardiogenic shock [21].

The GRACE score, which is recommended by clinical guidelines for risk stratification in acute coronary syndrome [8,22], was calculated for each patient to enable comparison with the nomogram developed in this study.

### End Point and Follow-Up

The primary end point was the occurrence of MARCE during hospitalization. MARCE was defined as a composite end point comprising all-cause mortality, acute or worsening renal failure requiring new renal replacement therapy (dialysis), new or recurrent AMI, and stroke [7]. Follow-up started at admission and ended at discharge or at in-hospital death.

### Feature Selection and Data Preprocessing

The initial dataset comprised 65 clinical variables. Among these, 59 features with missing data ≤10% were retained and imputed using the Multivariate Imputation by Chained Equations (MICE) package (version 3.13.0; Stef van Buuren and Karin Groothuis-Oudshoorn) in R software (version 4.1.0; R Foundation for Statistical Computing), with predictive mean matching [23]. To identify the most relevant predictors, least absolute shrinkage and selection operator (LASSO) regression with 10-fold cross-validation was applied based on the minimum binomial deviance criterion in the training set [24].

Given the wide numerical range across features and the requirement of certain ML algorithms for normalized inputs, min-max scaling was applied to continuous variables, and multicategory variables were processed using 1-hot encoding.

### Model Development

Two model types were constructed: a nomogram and tree-based ML models. The discovery cohort was randomly divided into a training set (375/536, 70%) and a test set (161/536, 30%). To ensure robustness and minimize bias, 10-fold cross-validation was applied during model training [25]. Each fold was created via stratified sampling to maintain the outcome distribution. Feature selection and model tuning were performed only on the training data, and final model evaluation was conducted on the untouched test set. Specifically, LASSO feature selection was performed once on the entire training set before cross-validation. The selected predictors were then used for subsequent model training, hyperparameter tuning, internal cross-validation, hold-out test evaluation, and external validation. The hold-out test set and external validation cohort were not used during feature selection or hyperparameter tuning.

### ML Algorithms and Interpretation

We used decision tree (DT), random forest (RF), and extreme gradient boosting (XGBoost) as representative tree-based algorithms. DT segments the feature space via hierarchical splits but is prone to overfitting and ignores feature interactions. RF combines multiple trees via bagging and random feature selection to improve accuracy and generalizability [26]. XGBoost enhances performance through regularized gradient boosting, parallel processing, and efficient feature handling [27].

Seven predictors identified by LASSO (Killip class, lactate, eGFR, cardiogenic shock, beta-blockers, ACEI/ARB/ARNI, and diuretics) were examined in multivariable modeling. Because Killip class IV is clinically defined as cardiogenic shock, these 2 variables represent substantial conceptual overlap. To improve clinical interpretability and reduce redundancy, we retained Killip class as the integrated severity indicator in the final parsimonious model and did not retain cardiogenic shock as a separate predictor after adjustment in the logistic regression (LR). Therefore, the final 6-variable predictor set should be considered a clinically refined model based on LASSO-selected predictors rather than a model derived exclusively through a fully data-driven feature-selection process.

We then trained XGBoost, RF, DT, and LR models using the same 6 clinically interpretable predictors. To interpret model predictions, SHAP values were used to quantify feature importance and to visualize both global and local contributions [28].

### Nomogram Construction

A nomogram was developed using multivariable LR based on the 6 selected features. To facilitate clinical application, an online calculator was created for visualization and risk estimation.

### Model Evaluation

All models were evaluated using 10-fold cross-validation on the training set and tested on the hold-out test set. The nomogram was further validated in the external cohort. Discrimination was assessed using the area under the receiver operating characteristic curve (AUC). Additional metrics included the Matthews correlation coefficient, precision (positive predictive value), negative predictive value, *F*_1_-score, accuracy, sensitivity, and specificity. Because these classification metrics are threshold-dependent, they were interpreted together with receiver operating characteristic and calibration performance. Calibration was assessed using the Brier score and visualized using calibration curves with 400 bootstrap samples. Decision curve analysis (DCA) and clinical impact curves were used to assess clinical utility and net benefit. For classification metrics (accuracy, *F*_1_-score, sensitivity, specificity, positive predictive value, and negative predictive value), a fixed probability classification threshold of 0.50 was used across all models, with MARCE as the positive class.

Two post hoc sensitivity analyses were conducted. First, the XGBoost model was refitted using conventional learning-rate values of 0.05 and 0.10 to assess whether model performance was dependent on the extremely small automatically tuned learning rate. Second, XGBoost, RF, DT, and LR models were reconstructed using all 7 LASSO-selected predictors, including cardiogenic shock. All sensitivity analyses used the original training-test split, and final evaluation was performed in the untouched held-out test set.

### Statistical Analysis

Continuous variables are described as means (SD) and categorical variables as frequencies and proportions. The clinical characteristics of patients were compared using the 2-tailed Student *t* test, Wilcoxon test, chi-square test, or Fisher exact probability method as appropriate. The evaluation indices of the ML models were compared using 1-way ANOVA with multiple comparisons of the least-significant difference. A 2-tailed *P* value <.05 was considered statistically significant. The statistical analyses were performed using R (version 4.1.0 [29]). AUC comparisons between models were additionally assessed using the DeLong test.

## Results

### Study Population

The study flowchart is shown in Figure 1. From January 2013 to July 2019, a total of 536 patients were enrolled in the discovery cohort. The mean (SD) of their age was 72.83 (10.31) years, and 372 (69.4%) of the participants were male. Of them, 18.1% (n=97) died in the hospital, 8.6% (n=46) had renal failure necessitating new renal replacement therapy, 3.2% (n=17) had a new or recurrent myocardial infarction (MI), and 1.7% (n=9) had a stroke. They were randomly assigned to a training set (n=375) or a test set (n=161). There were no significant differences in the patient characteristics of the 2 datasets (Table 1). The rates of MARCE were 24.8% (n=133) in the train and test sets. In both sets, differences in clinical characteristics between patients who had MARCE and those who did not are outlined in Table 2. Data from the validation set were obtained and analyzed on 157 patients collected from Wenzhou TCM Hospital of Zhejiang Chinese Medical University, including 35 (22.3%) cases of MARCE (Table S1 in Multimedia Appendix 1).

**Figure 1.**
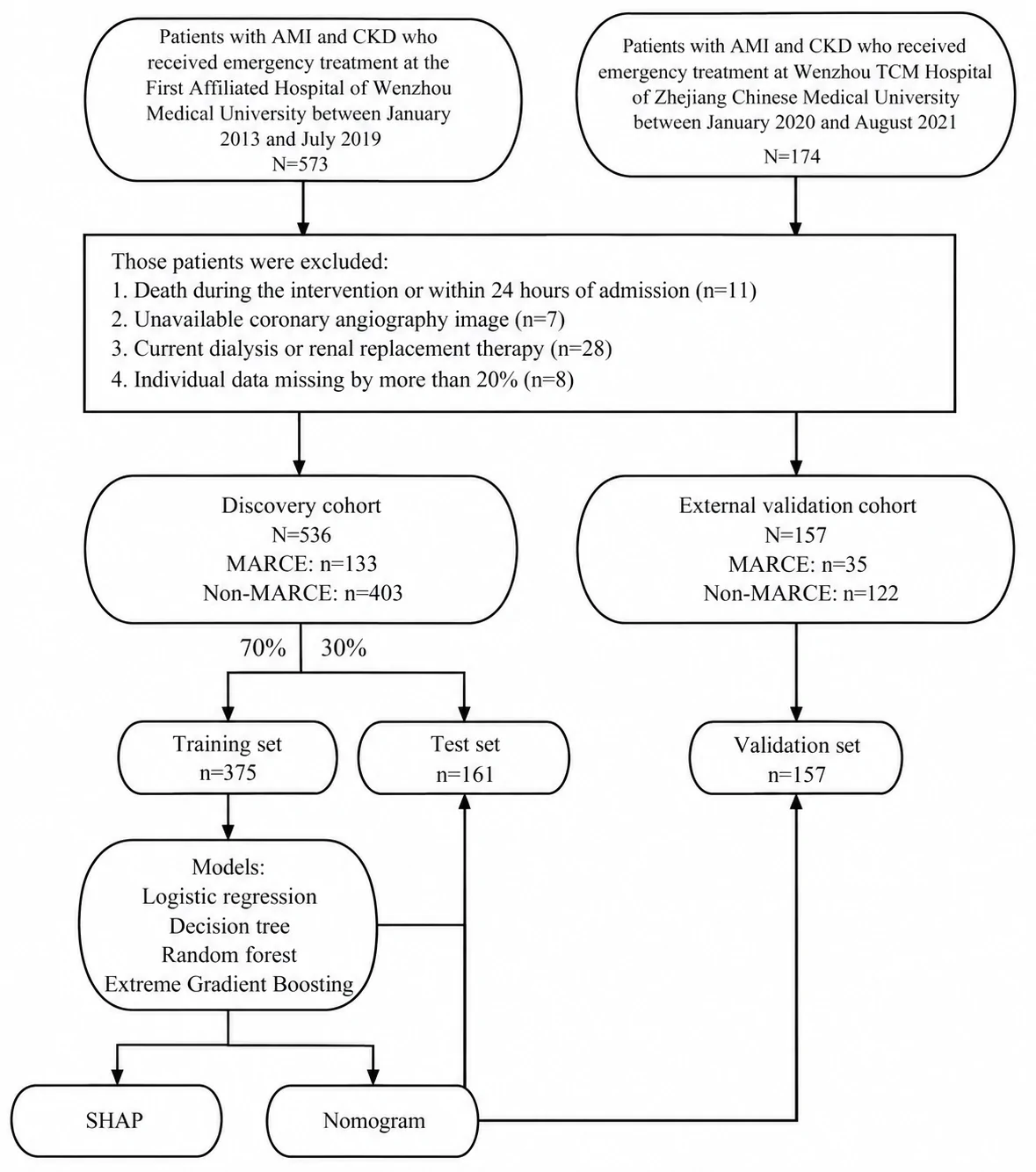
Formation of the discovery and validation cohorts and occurrence of major adverse renal and cardiovascular events in patients with acute myocardial infarction (AMI) and chronic kidney disease (CKD). MARCE: major adverse renal and cardiovascular events; SHAP: Shapley additive explanations; TCM: traditional Chinese medicine; XGBoost: extreme gradient boosting.

**Table 1.** Clinical characteristics of patients in the discovery set.^a^

Variables	Training set (N=375)	Test set (N=161)	*P* value
Demographic			
Age (y), mean (SD)	73.05 (10.38)	72.31 (10.14)	.45
Sex, n (%)			.20
Female	121 (32.27)	43 (26.71)	
Male	254 (67.73)	118 (73.29)	
Presentation characteristics			
ST-segment elevation, n (%)			.60
0	54 (14.4)	26 (16.15)	
1	321 (85.6)	135 (83.85)	
Killip class, n (%)			.79
1	178 (47.47)	82 (50.93)	
2	62 (16.53)	24 (14.91)	
3	17 (4.53)	9 (5.59)	
4	118 (31.47)	46 (28.57)	
Cardiogenic shock, n (%)			.72
0	310 (82.67)	131 (81.37)	
1	65 (17.33)	30 (18.63)	
Syncope, n (%)			.28
0	342 (91.2)	142 (88.2)	
1	33 (8.8)	19 (11.80)	
Tracheal cannula, n (%)			.07
0	354 (94.4)	145 (90.06)	
1	21 (5.6)	16 (9.94)	
Cardiopulmonary resuscitation, n (%)			.80
0	356 (94.93)	152 (94.41)	
1	19 (5.07)	9 (5.59)	
Heart rate (beats/min), mean (SD)	88.4 (37.6)	82.64 (19.79)	.07
Systolic blood pressure (mm Hg), mean (SD)	120.8 (24.33)	117.16 (24.16)	.11
Diastolic blood pressure (mm Hg), mean (SD)	71.14 (15.46)	68.92 (15.13)	.13
Procedural characteristics, n (%)			
LM^b^ stenosis ≥75			.49
0	353 (94.13)	149 (92.55)	
1	22 (5.87)	12 (7.45)	
LAD^c^ stenosis ≥75			.77
0	126 (33.6)	52 (32.3)	
1	249 (66.4)	109 (67.7)	
LCX^d^ stenosis ≥75			.64
0	246 (65.6)	109 (67.7)	
1	129 (34.4)	52 (32.3)	
RCA^e^ stenosis ≥75			.43
0	163 (43.47)	64 (39.75)	
1	212 (56.53)	97 (60.25)	
Multivessel disease			.62
0	315 (84)	138 (85.71)	
1	60 (16)	23 (14.29)	
Calcification			.04
0	340 (90.67)	136 (84.47)	
1	35 (9.33)	25 (15.53)	
Thrombus			.14
0	219 (58.4)	83 (51.55)	
1	156 (41.6)	78 (48.45)	
Thrombus aspiration			.62
0	262 (69.87)	109 (67.7)	
1	113 (30.13)	52 (32.3)	
Medication at admission, n (%)			
Aspirin			.51
0	1 (0.27)	1 (0.62)	
1	374 (99.73)	160 (99.38)	
P2Y12 inhibitor			.51
0	1 (0.27)	1 (0.62)	
1	374 (99.73)	160 (99.38)	
Statin			.14
0	97 (25.87)	32 (19.88)	
1	278 (74.13)	129 (80.12)	
Beta-blockers			.82
0	187 (49.87)	82 (50.93)	
1	188 (50.13)	79 (49.07)	
ACEI^f^ /ARB^g^ /ARNI^h^			.85
0	218 (58.13)	95 (59.01)	
1	157 (41.87)	66 (40.99)	
Diuretics			.17
0	215 (57.33)	82 (50.93)	
1	160 (42.67)	79 (49.07)	
Laboratory data			
Lactate (mmol/L), median (IQR)	2.8 (1.9-4.5)	2.7 (1.9-4.5)	.89
Troponin (ng/mL), median (IQR)	22.85 (2.58-50)	29.18 (2.17-50)	.48
CK^i^ (U/L), median (IQR)	1034 (260.5-3718)	1012(334-4074)	.63
CK MB^j^ (ng/mL), median (IQR)	108 (37-277)	117 (35-294)	.89
BNP^k^ (pg/mL), median (IQR)	288 (90.5-872.5)	280 (77-670)	.28
Creatinine (μmol/L), mean (SD)	120.71 (66.19)	116.31 (55.54)	.46
eGFR^l^ (mL/min/1.73 m^2^), mean (SD)	38.53 (14.87)	39.44 (14.44)	.51
Uric acid (μmol/L), mean (SD)	479.55 (161.8)	459.75 (141.87)	.18
ALT^m^ (U/L), median (IQR)	56 (33-105)	62 (34-108)	.63
AST^n^ (U/L), median (IQR)	266 (109-476.5)	255 (114-547)	.85
Total cholesterol (mmol/L), mean (SD)	4.89 (3.11)	4.85 (1.53)	.88
Triglyceride (mmol/L), mean (SD)	1.64 (2.6)	1.67 (1.4)	.88
LDL-C^o^ (mmol/L), mean (SD)	2.93 (1.17)	2.98 (1.1)	.66
** **HDL-C^p^ (mmol/L), mean (SD)	1 (0.27)	0.99 (0.26)	.71
FBG^q^ (mmol/L), mean (SD)	8.88 (4.52)	8.92 (3.96)	.92
Potassium (mmol/L), mean (SD)	4.3 (0.7)	4.28 (0.62)	.78
CRP^r^ (μg/L), median (IQR)	35.3 (11.65-76.4)	28.2 (8-66.2)	.10
T3^s^ (nmol/L), mean (SD)	0.97 (0.34)	0.92 (0.31)	.11
FT3^t^ (pmol/L), mean (SD)	3.95 (0.79)	3.85 (0.75)	.18
FT4^u^ (pmol/L), mean (SD)	12.36 (3.17)	11.98 (2.6)	.18
TSH^v^ (mIU/L), median (IQR)	1.03 (0.58-1.83)	0.93 (0.52-1.72)	.34
D-dimer (mg/L), median (IQR)	1.32 (0.84-2.79)	1.54 (0.97-2.89)	.23
Hemoglobin (g/L), mean (SD)	121.24 (20.49)	122.4 (18.68)	.54
Fibrinogen (g/L), mean (SD)	4.06 (1.4)	3.88 (1.33)	.16
WBC^w^ (×10^9^ /L), mean (SD)	13.24 (5.33)	13.62 (6.08)	.50
LVEF^x^ (%), mean (SD)	45.13 (10.3)	46.28 (10.47)	.24
Medical history, n (%)			
Previous CAD^y^			.41
0	344 (91.73)	151 (93.79)	
1	31 (8.27)	10 (6.21)	
Previous stroke			.31
0	334 (89.07)	148 (91.93)	
1	41 (10.93)	13 (8.07)	
Previous cerebral hemorrhage			.51
0	374 (99.73)	160 (99.38)	
1	1 (0.27)	1 (0.62)	
Hypertension			.85
0	122 (32.53)	51 (31.68)	
1	253 (67.47)	110 (68.32)	
Diabetes			.20
0	259 (69.07)	120 (74.53)	
1	116 (30.93)	41 (25.47)	
Hyperlipidemia			.92
0	332 (88.53)	143 (88.82)	
1	43 (11.47)	18 (11.18)	
Current smoking			.09
0	261 (69.6)	100 (62.11)	
1	114 (30.4)	61 (37.89)	
Current drinking			.01
0	320 (85.33)	123 (76.4)	
1	55 (14.67)	38 (23.6)	
Outcome, n (%)			
MARCE^z^			.99
0	282 (75.2)	121 (75.16)	
1	93 (24.8)	40 (24.84)	

a Continuous variables are presented as mean (SD) for normally distributed variables or median (IQR) for nonnormally distributed variables, while categorical variables are presented as number (percentage).

b LM: left main disease.

c LAD: left anterior descending coronary.

d LCX: left circumflex artery.

e RCA: right coronary artery.

f ACEI: angiotensin-converting enzyme inhibitor.

g ARB: angiotensin II receptor blocker.

h ARNI: angiotensin receptor-neprilysin inhibitor.

i CK: creatine kinase.

j CK MB: creatine kinase myocardial band.

k BNP: brain natriuretic peptide.

l eGFR: estimated glomerular filtration rate.

m ALT: alanine aminotransferase.

n AST: aspartate aminotransferase.

o LDL-C: low-density lipoprotein cholesterol level.

p HDL-C: high-density lipoprotein cholesterol level.

q FBG: fasting blood glucose.

r CRP: C reactive protein.

s T3: triiodothyronine.

t FT3: free triiodothyronine.

u FT4: free thyroxine.

v TSH: thyroid stimulating hormone.

w WBC: white blood cell.

x LVEF: left ventricular ejection fraction.

y CAD: coronary heart disease.

z MARCE: major adverse renal and cardiovascular events.

**Table 2.** Clinical characteristics of patients who did or did not develop MARCE^a^ in the discovery set.^b^

Variables	No MARCE (N=403)	MARCE (N=133)	*P* value
Demographic			
Age (y), mean (SD)	72.6 (10.48)	73.53 (9.78)	.37
Sex, n (%)			.03
Female	113 (28.04)	51 (38.35)	
Male	290 (71.96)	82 (61.65)	
Presentation characteristics			
ST segment elevation, n (%)			.01
0	51 (12.66)	29 (21.8)	
1	352 (87.34)	104 (78.2)	
Killip class, n (%)			<.001
1	236 (58.56)	24 (18.05)	
2	73 (18.11)	13 (9.77)	
3	18 (4.47)	8 (6.02)	
4	76 (18.86)	88 (66.17)	
Cardiogenic shock, n (%)			<.001
0	362 (89.83)	79 (59.4)	
1	41 (10.17)	54 (40.6)	
Syncope, n (%)			.30
0	367 (91.07)	117 (87.97)	
1	36 (8.93)	16 (12.03)	
Tracheal cannula, n (%)			<.001
0	389 (96.53)	110 (82.71)	
1	14 (3.47)	23 (17.29)	
Cardiopulmonary resuscitation, n (%)			.17
0	385 (95.53)	123 (92.48)	
1	18 (4.47)	10 (7.52)	
Heart rate (beats/min), mean (SD)	83.98 (35.1)	94.82 (25.86)	.001
Systolic blood pressure (mm Hg), mean (SD)	121.39 (23.02)	114.6 (27.33)	.01
Diastolic blood pressure (mm Hg), mean (SD)	71.31 (14.63)	67.93 (17.26)	.03
Procedural characteristics, n (%)			
LM^c^ stenosis ≥75			.14
0	381 (94.54)	121 (90.98)	
1	22 (5.46)	12 (9.02)	
LAD^d^ stenosis ≥75			.65
0	136 (33.75)	42 (31.58)	
1	267 (66.25)	91 (68.42)	
LCX^e^ stenosis ≥75			.02
0	278 (68.98)	77 (57.89)	
1	125 (31.02)	56 (42.11)	
RCA^f^ stenosis ≥75			.25
0	165 (40.94)	62 (46.62)	
1	238 (59.06)	71 (53.38)	
Multivessel disease			.22
0	345 (85.61)	108 (81.2)	
1	58 (14.39)	25 (18.8)	
Calcification			.11
0	363 (90.07)	113 (84.96)	
1	40 (9.93)	20 (15.04)	
Thrombus			.10
0	219 (54.34)	83 (62.41)	
1	184 (45.66)	50 (37.59)	
Thrombus aspiration			.053
0	270 (67)	101 (75.94)	
1	133 (33)	32 (24.06)	
Medication at admission, n (%)			
Aspirin			>.99
0	2 (0.50)	0 (0)	
1	401 (99.50)	133 (100)	
P2Y12 inhibitor			>.99
0	2 (0.50)	0 (0)	
1	401 (99.50)	133 (100)	
Statin			.81
0	98 (24.32)	31 (23.31)	
1	305 (75.68)	102 (76.69)	
Beta-blockers			<.001
0	158 (39.21)	111 (83.46)	
1	245 (60.79)	22 (16.54)	
ACEI^g^ /ARB^h^ /ARNI^i^			<.001
0	198 (49.13)	115 (86.47)	
1	205 (50.87)	18 (13.53)	
Diuretics			<.001
0	192 (47.64)	105 (78.95)	
1	211 (52.36)	28 (21.05)	
Laboratory data			
Lactate (mmol/L), median (IQR)	2.4 (1.7-3.6)	4.5 (2.6-8.8)	<.001
Troponin (ng/mL), median (IQR)	24.07 (2.81-50)	25.98 (1.34-50)	.34
CK^j^ (U/L), median (IQR)	1033 (315.5-3840.5)	1061 (240-3763)	.68
CK MB^k^ (ng/mL), median (IQR)	109 (38-292)	119 (30-277)	.64
BNP^l^ (pg/mL), median (IQR)	256 (81-707)	428 (112-1175)	.004
Creatinine (μmol/L), mean (SD)	120.71 (66.19)	116.31 (55.54)	.46
eGFR^m^ (mL/min/1.73 m^2^), mean (SD)	38.53 (14.87)	39.44 (14.44)	.51
Uric acid (μmol/L), mean (SD)	464.88 (140.71)	500.04 (194.09)	.06
ALT^n^ (U/L), median (IQR)	54 (32-93)	80 (35-171)	.001
AST^o^ (U/L), median (IQR)	233 (107.5-426.5)	399 (119-994)	<.001
Total cholesterol (mmol/L), mean (SD)	4.77 (1.42)	5.2 (4.89)	.32
Triglyceride (mmol/L), mean (SD)	1.53 (1.1)	2 (4.2)	.20
LDL C^p^ (mmol/L), mean (SD)	2.92 (1.08)	3.02 (1.33)	.45
HDL C^q^ (mmol/L), mean (SD)	1 (0.26)	0.99 (0.31)	.92
FBG^r^ (mmol/L), mean (SD)	8.34 (3.8)	10.57 (5.39)	<.001
Potassium (mmol/L), mean (SD)	4.26 (0.6)	4.4 (0.84)	.07
CRP^s^ (μg/L), median (IQR)	28.7 (9.3-69.1)	53.2 (15.7-89.9)	.006
T3^t^ (nmol/L), mean (SD)	0.99 (0.32)	0.87 (0.35)	.001
FT3^u^ (pmol/L), mean (SD)	3.99 (0.78)	3.71 (0.76)	<.001
FT4^v^ (pmol/L), mean (SD)	12.08 (2.85)	12.76 (3.43)	.02
TSH^w^ (mIU/L), mean (SD)	1.79 (4.31)	2.33 (5.87)	.26
D-dimer (mg/L), median (IQR)	1.27 (0.82-2)	2.08 (1.03-6.02)	<.001
Hemoglobin (g/L), mean (SD)	122.03 (18.76)	120.24 (23.24)	.42
Fibrinogen (g/L), mean (SD)	3.93 (1.27)	4.25 (1.66)	.04
WBC^x^ (×10^9^ /L), mean (SD)	12.42 (4.89)	16.2 (6.46)	<.001
LVEF^y^ (%), mean (SD)	46.8 (9.94)	41.46 (10.58)	<.001
Medical history, n (%)			
Previous CAD^z^			.29
0	375 (93.05)	120 (90.23)	
1	28 (6.95)	13 (9.77)	
Previous stroke			.13
0	367 (91.07)	115 (86.47)	
1	36 (8.93)	18 (13.53)	
Previous cerebral hemorrhage			.44
0	402 (99.75)	132 (99.25)	
1	1 (0.25)	1 (0.75)	
Hypertension			.28
0	125 (31.02)	48 (36.09)	
1	278 (68.98)	85 (63.91)	
Diabetes			.12
0	292 (72.46)	87 (65.41)	
1	111 (27.54)	46 (34.59)	
Hyperlipidemia			.053
0	351 (87.1)	124 (93.23)	
1	52 (12.9)	9 (6.77)	
Current smoking			.17
0	265 (65.76)	96 (72.18)	
1	138 (34.24)	37 (27.82)	
Current drinking			.42
0	330 (81.89)	113 (84.96)	
1	73 (18.11)	20 (15.04)	

a MARCE: major adverse renal and cardiovascular events.

b Continuous variables are presented as mean (SD) for normally distributed variables or median (IQR) for nonnormally distributed variables, while categorical variables are presented as number (percentage).

c LM: left main disease.

d LAD: left anterior descending coronary.

e LCX: left circumflex artery.

f RCA: right coronary artery.

g ACEI: angiotensin-converting enzyme inhibitor.

h ARB: angiotensin II receptor blocker.

i ARNI: angiotensin receptor-neprilysin inhibitor.

j CK: creatine kinase.

k CK MB: creatine kinase myocardial band.

l BNP: brain natriuretic peptide.

m eGFR: estimated glomerular filtration rate.

n ALT: alanine aminotransferase.

o AST: aspartate aminotransferase.

p LDL-C: low-density lipoprotein cholesterol level.

q HDL-C: high-density lipoprotein cholesterol level.

r FBG: fasting blood glucose.

s CRP: C reactive protein.

t T3: triiodothyronine.

u FT3: free triiodothyronine.

v FT4: free thyroxine.

w TSH: thyroid stimulating hormone.

x WBC: white blood cell.

y LVEF: left ventricular ejection fraction.

z CAD: coronary heart disease.

### Feature Selection

Among the 59 demographic details, comorbidities, laboratory results, echocardiography measurements, procedural details, and medication parameters, 7 significant predictors of MARCE were selected in the training set by the LASSO algorithm (Figure 2A and B): Killip class, lactate, eGFR, cardiogenic shock, beta-blockers, ACEI/ARB/ARNI, and diuretics. Given that Killip class IV clinically corresponds to cardiogenic shock, we retained Killip class as the broader, clinically interpretable severity variable and excluded cardiogenic shock from the final parsimonious nomogram to avoid redundant representation of overlapping information. The multivariable analysis of the 7 LASSO-selected predictors is presented in Table 3. After excluding cardiogenic shock due to clinical redundancy with Killip class, the final 6 predictors were used for model development.

**Figure 2.**
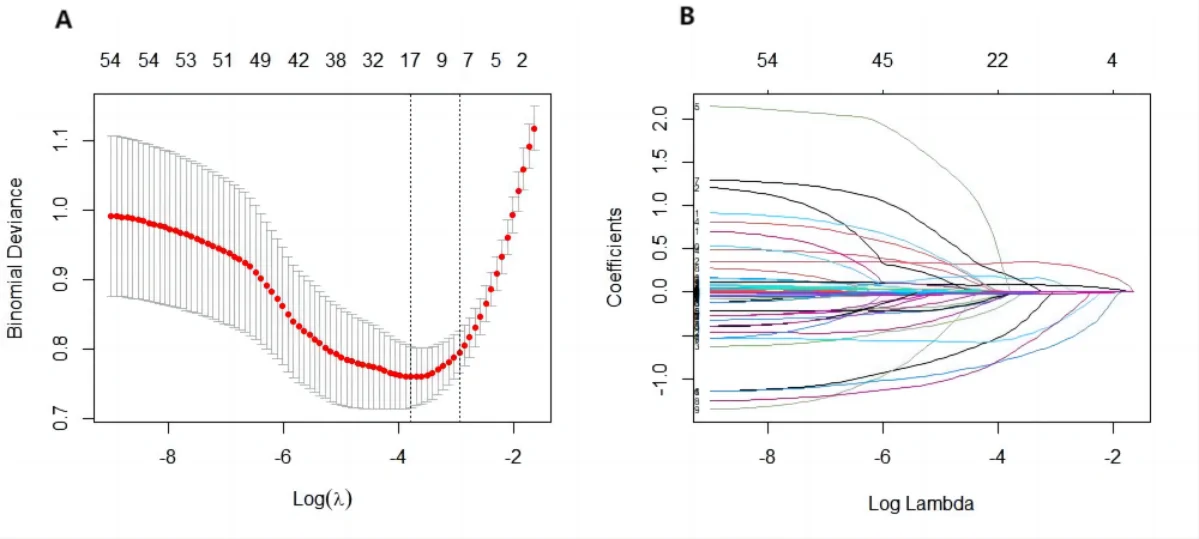
Feature selection using the least absolute shrinkage and selection operator (LASSO) model. (A) Selection of the tuning parameter (λ) in the LASSO model via 10-fold cross-validation based on the 1-SE criterion. Binomial deviance from the LASSO cross-validation procedure was plotted as a function of log(λ). The y-axis indicates binomial deviance, and the x-axis indicates log(λ). Numbers along the upper x-axis represent the average number of predictors. Red dots indicate average deviance values for each model with a given λ, and vertical bars show upper and lower deviance values. The vertical black lines define the optimal values of λ. (B) LASSO coefficient profiles of clinical features. The dotted vertical line indicates the selected λ. Seven features with nonzero coefficients are shown.

**Table 3.** Multivariate logistic regression analysis of risk factors for MARCE^a^ in the training set.

Variables	B	SE	*z*	*P* value	OR^b^ (95% CI)
MARCE					
Constant	0.045	0.481	0.093	.93	—^c^
Killip class, (reference: 1)					
2	0.437	0.418	1.046	.30	1.55 (0.68-3.51)
3	0.983	0.573	1.714	.09	2.67 (0.87-8.22)
4	1.302	0.350	3.723	<.001	3.68 (1.85-7.3)
Cardiogenic shock	0.511	0.342	1.495	.14	1.67 (0.85-3.26)
Use of beta-blockers	–0.968	0.307	–3.155	.002	0.38 (0.21-0.69)
Use of ACEI^d^ /ARB^e^ /ARNI^f^	–0.838	0.325	–2.576	.01	0.43 (0.23-0.82)
Use of diuretics	–0.97	0.299	–3.244	.001	0.38 (0.21-0.68)
Lactate	0.106	0.049	2.169	.03	1.11 (1.01-1.22)
eGFR^g^	–0.038	0.009	–4.014	<.001	0.96 (0.95-0.98)

a MARCE: major adverse renal and cardiovascular events.

b OR: odds ratio.

c Not applicable.

d ACEI: angiotensin-converting enzyme inhibitor.

e ARB: angiotensin II receptor blocker.

f ARNI: angiotensin receptor-neprilysin inhibitor.

g eGFR: estimated glomerular filtration rate.

### ML Models

Six selected variables were included in the following ML models: XGBoost, RF, DT, and LR. Figure 3A and Table 4 illustrate model performance in the test set. XGBoost achieved an AUC of 0.882 (95% CI 0.816‐0.947), LR 0.875 (95% CI 0.799‐0.950), RF 0.870 (95% CI 0.800‐0.940), and DT 0.814 (95% CI 0.720‐0.886). Given the overlap in confidence intervals, these findings indicate comparable discrimination among XGBoost, LR, and RF rather than a definitive ranking of model superiority. Ten-fold cross-validation results for AUC and accuracy in the training set are summarized in Table S2 in Multimedia Appendix 1. Calibration plots showed acceptable calibration for all models (Figure 3B, Table 4). Other evaluation indicators are summarized in Table 4. Confusion matrices for all 4 classifiers in the test set are shown in Table S3 in Multimedia Appendix 1. Pairwise AUC comparisons showed no statistically significant differences among XGBoost, LR, and RF (XGBoost vs LR: *P*=.89; XGBoost vs RF: *P*=.81; LR vs RF: *P*=.92). Comparisons against DT were also not significant (all *P*>.05; Table S4 in Multimedia Appendix 1).

**Figure 3.**
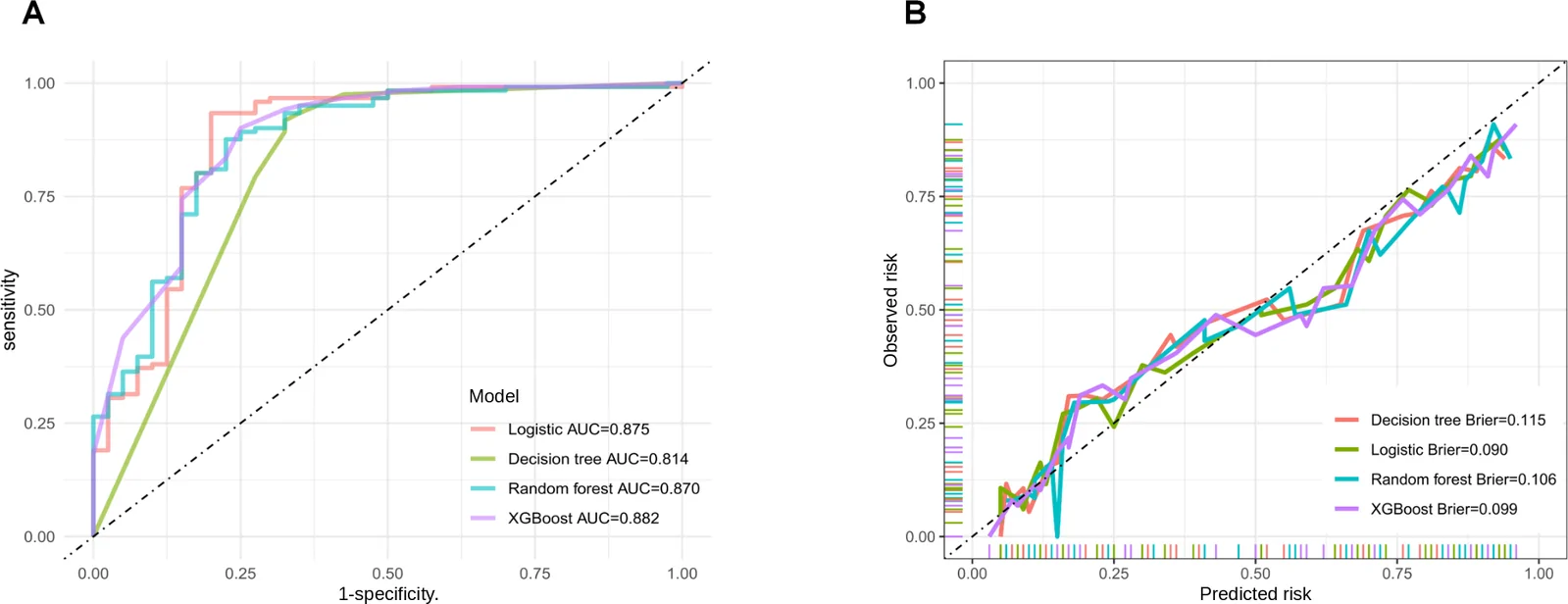
Performance of machine learning models in the test set. (A) Comparison of area under the receiver operating characteristic curves among extreme gradient boosting (XGBoost), random forest, logistic regression, and decision tree models in the test set. (B) Calibration plots of XGBoost, random forest, logistic regression, and decision tree models in the test set. The Brier score is reported in the lower right legend (the smaller the value, the better the calibration). AUC: area under the receiver operating characteristic curve.

**Table 4.** Classifiers’ predictive performance in the test set.^a^

Index	Logistic	XGBoost^b^	Random forest	Decision tree
AUC^c^ (95% CI)	0.875 (0.799‐0.950)	0.882 (0.816‐0.947)	0.870 (0.800‐0.940)	0.814 (0.720‐0.886)
Brier score (95% CI)	0.090 (0.056‐0.124)	0.099 (0.065‐0.132)	0.106 (0.071‐0.140)	0.115 (0.077‐0.152)
Accuracy	0.888	0.807	0.857	0.863
MCC^d^	0.685	0.414	0.608	0.618
*F*_1_-score	0.743	0.392	0.701	0.703
κ	0.673	0.319	0.608	0.615
PPV^e^	0.867	0.909	0.730	0.765
NPV^f^	0.893	0.800	0.895	0.890
Sensitivity	0.650	0.250	0.675	0.650
Specificity	0.967	0.992	0.917	0.934

a Classification metrics were calculated using major adverse renal and cardiovascular events (MARCE) as the positive class at a fixed threshold of 0.50.

b XGBoost: extreme gradient boosting.

c AUC: area under the receiver operating characteristic curve.

d MCC: Matthews correlation coefficient.

e PPV: positive predictive value.

f NPV: negative predictive value.

Post hoc sensitivity analyses were performed to assess the robustness of the XGBoost model and the final predictor set. Refitting XGBoost with conventional learning-rate values of 0.05 and 0.10 yielded similar discrimination compared with the originally tuned model, with AUCs of 0.876 and 0.870, respectively, vs 0.882 for the original model (Multimedia Appendix 2). Additionally, models reconstructed using all 7 LASSO-selected predictors, including cardiogenic shock, showed comparable performance to the final 6-variable models. These findings suggest that the main results were not materially dependent on the extremely small tuned XGBoost learning rate or on the exclusion of cardiogenic shock (Tables S5 and S6 in Multimedia Appendix 1).

### SHAP Values Through Visualization

Comparisons among the 4 ML-based models for MARCE prediction showed that XGBoost achieved the numerically highest AUC in the test set, although its performance was comparable to LR and RF rather than being definitively superior. The final hyperparameters for all classifiers are provided in Table S7 in Multimedia Appendix 1.

We used SHAP values to provide feature attribution values for each variable within the XGBoost model. Figure 4A describes the SHAP summary plot for the 6 input variables in the training set. The y-axis displays the 6 variables, ranked in order of importance by their mean absolute SHAP values. The x-axis indicates the SHAP values associated with each variable and patient, which allowed the determination of whether a feature had a negative effect on the prediction toward a non-MARCE class or a positive effect on the prediction toward a MARCE class (Figure 4B).

**Figure 4.**
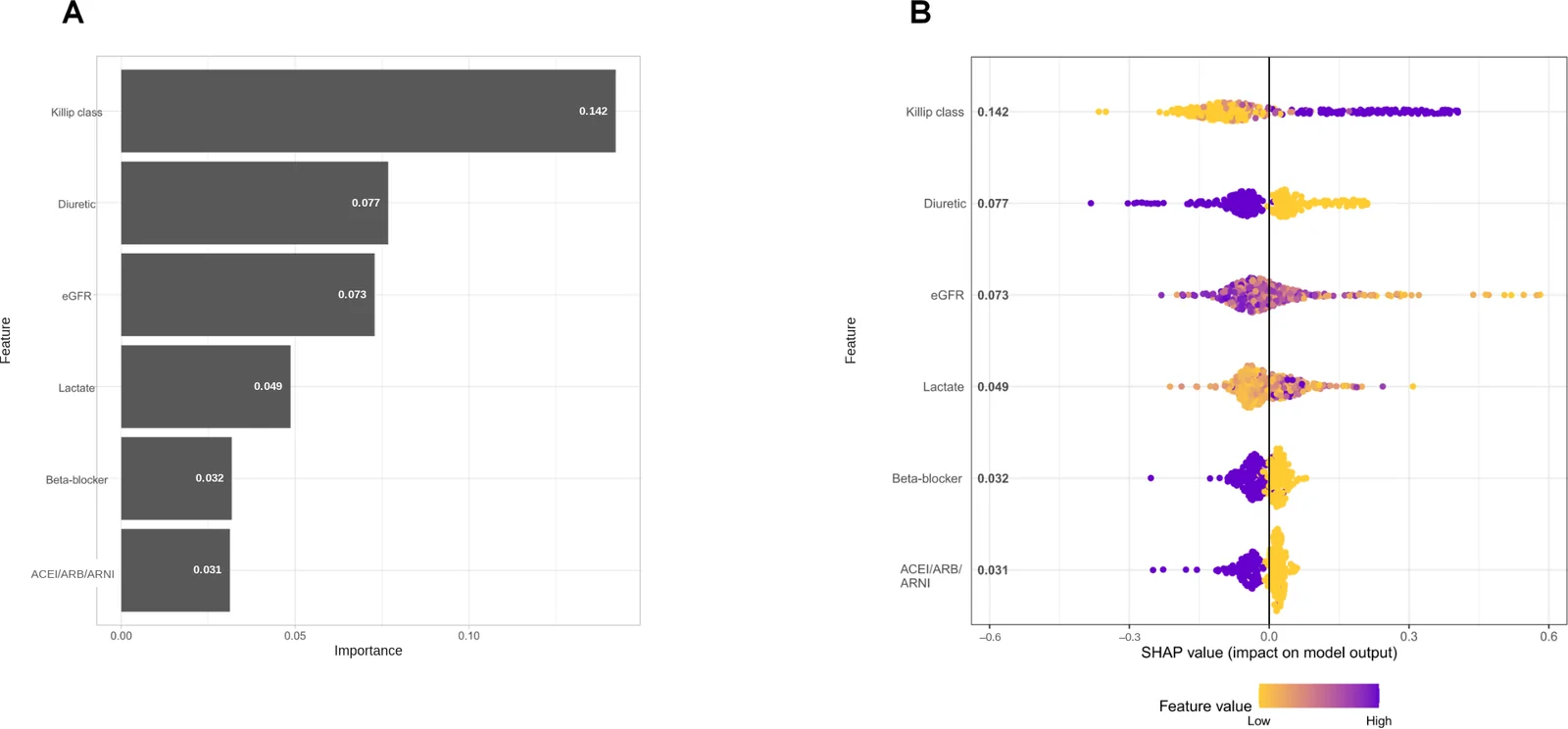
Shapley additive explanations (SHAP) summary plot of the extreme gradient boosting (XGBoost) model with selected features. (A) Average absolute impact of variables on the magnitude of the final model output, ordered by decreasing feature importance. (B) The plot depicts the dot estimates on the model output for the XGBoost model. Each dot represents an individual patient from the dataset. Purple represents higher SHAP values of specific features, whereas yellow represents lower SHAP values of specific features. Higher SHAP values indicate a greater risk of major adverse renal and cardiovascular events. ACEI: angiotensin-converting enzyme inhibitor; ARB: angiotensin receptor blocker; ARNI: angiotensin receptor-neprilysin inhibitor; eGFR: estimated glomerular filtration rate.

Beyond ranking feature importance, SHAP analysis provided a patient-level attribution of predicted MARCE risk. The Killip class had the largest mean absolute SHAP value, indicating that hemodynamic severity was the dominant contributor to the model output. Diuretic use, eGFR, and lactate also contributed substantially, suggesting that volume overload or heart failure treatment status, renal dysfunction, and metabolic stress jointly influenced MARCE risk prediction.

### Establishment and External Validation of the Nomogram Model

The 6 selected predictors were incorporated to form a MARCE estimation nomogram (Figure 5). We compared the performance of the nomogram model with the GRACE score in the test set. The nomogram achieved an AUC of 0.875 (95% CI 0.800‐0.934), whereas the GRACE score achieved 0.765 (95% CI 0.675‐0.826) (Figure 6A and B). Both models showed acceptable calibration in the test set, with mean absolute error values of 0.047 and 0.045, respectively (Figure 6C and D). DCA suggested a potential net benefit of the nomogram across clinically relevant threshold ranges, rather than uniformly across all possible thresholds (Multimedia Appendix 3). In additional AUC comparison analysis, the nomogram showed higher discrimination than the GRACE score in this cohort (AUC difference=0.110, *P*=.03; Table S4 in Multimedia Appendix 1).

To further evaluate the nomogram, we analyzed an external validation cohort of 157 patients from Wenzhou TCM Hospital of Zhejiang Chinese Medical University. The nomogram displayed an AUC of 0.873 (95% CI 0.714‐0.926; Multimedia Appendix 4). Calibration plots with 400 bootstrap resampling confirmed agreement between predicted and observed risk (mean absolute error=0.042; Multimedia Appendix 4). DCA and clinical impact curve suggested potential clinical utility in the validation cohort (Multimedia Appendix 4), and these findings should be interpreted with caution because of the limited external sample size.

To facilitate clinical use, we developed a web-based interface for estimating the probability of in-hospital MARCE (Multimedia Appendix 5) [30].

**Figure 5.**
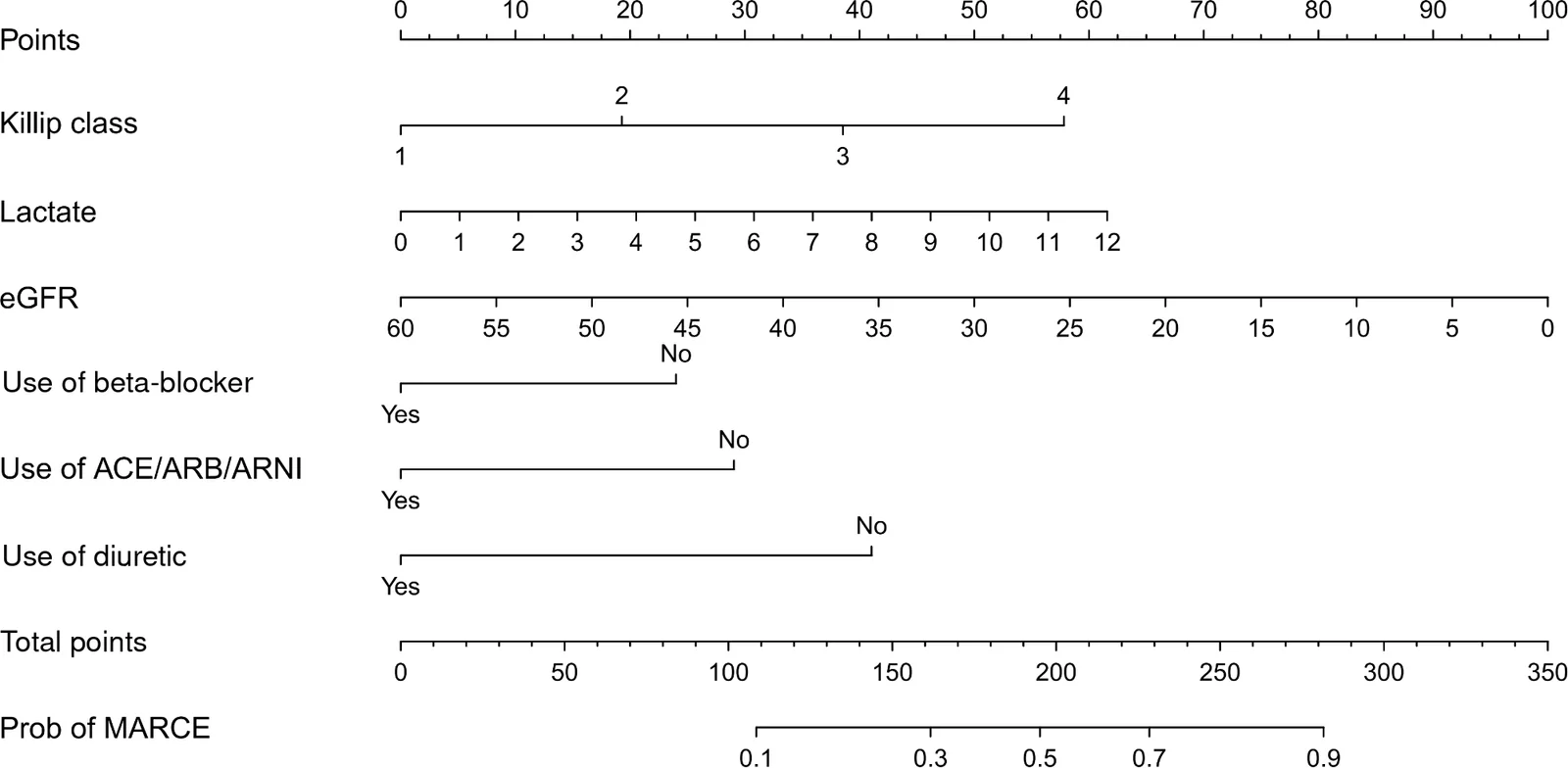
Nomogram predicting major adverse renal and cardiovascular events in patients with acute myocardial infarction and chronic kidney disease. ACE: angiotensin-converting enzyme; ARB: angiotensin receptor blocker; ARNI: angiotensin receptor-neprilysin inhibitor; eGFR: estimated glomerular filtration rate; MARCE: major adverse renal and cardiovascular events.

**Figure 6.**
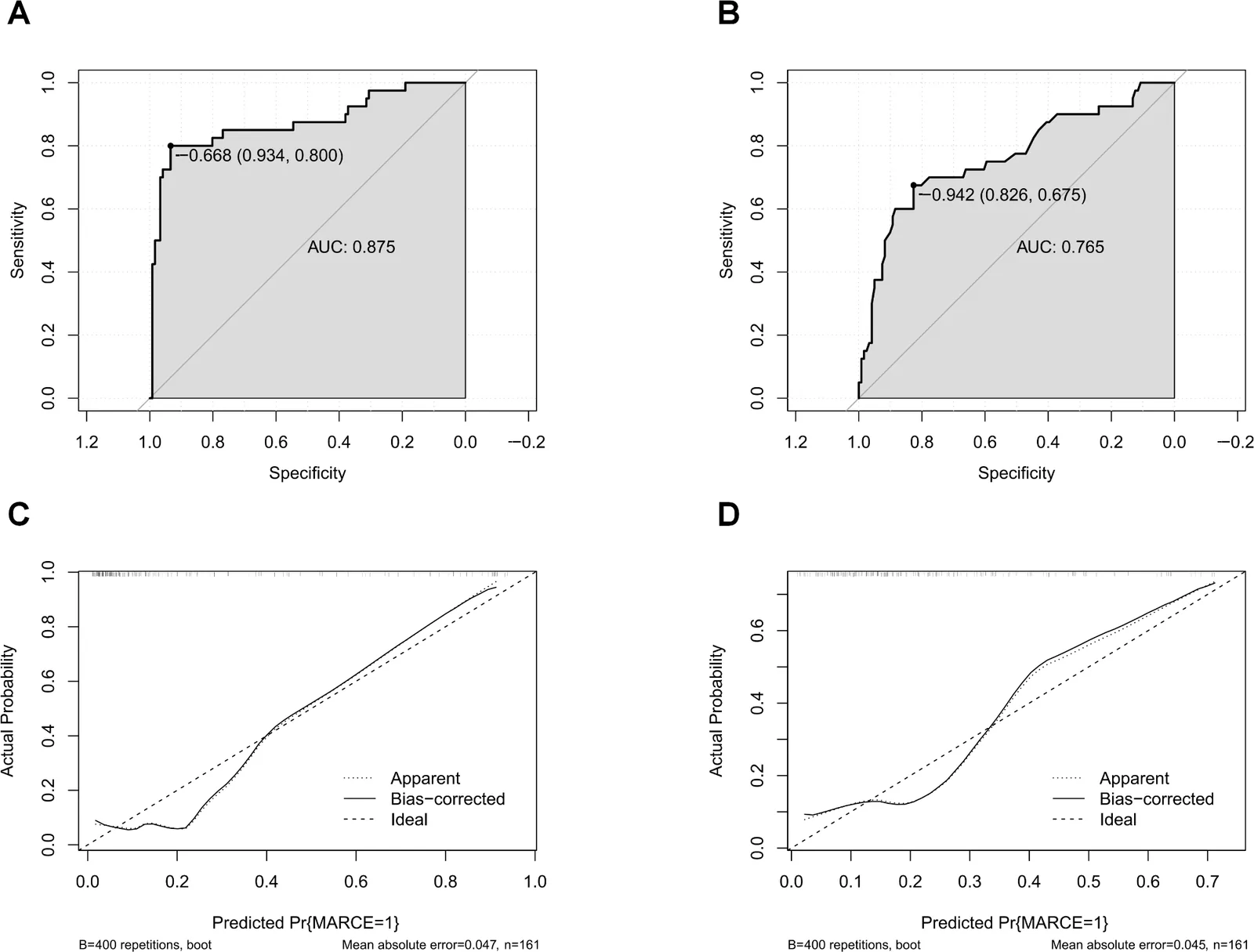
Performance comparison between the nomogram model and the Global Registry of Acute Coronary Events (GRACE) score in the test set. Receiver operating characteristic curve of the nomogram (A) and the GRACE score (B) in the test set; 400-resample bootstrapped calibration plots of the nomogram (C) and the GRACE score (D). AUC: area under the receiver operating characteristic curve; MARCE: major adverse renal and cardiovascular events.

## Discussion

### Principal Findings

In the present study, we applied LASSO-based feature screening and developed a series of models for in-hospital MARCE prediction in patients with AMI and CKD. We identified 6 selected predictors and observed moderate-to-good discrimination across ML and LR models (test set: AUC 0.814, 95% CI 0.720-0.886; AUC 0.882, 95% CI 0.816-0.947). SHAP analysis provided interpretable global and individual feature contributions. We also developed a nomogram and externally validated it, with acceptable discrimination and calibration. Given the close performance among XGBoost, LR, and RF, our results support a balanced emphasis on both predictive performance and clinical interpretability.

Previous research on adverse event prediction models has mostly focused on patients with AMI and excluded patients with CKD, thus reducing performance when applied to patients with CKD [10,11]. Troponin protein, which has been identified as a risk factor for adverse outcomes and is involved in many risk scores, can be cleared by the kidneys, thus further leading to the reduced predictive efficacy of such models in the population with CKD [31-33]. In a small cohort of 113 patients, Fu et al [34] developed a risk score using patient demographic and laboratory data to predict in-hospital mortality in patients with AMI and end-stage renal disease. The risk score calibrated well and performed better than the traditional GRACE scores, but the study population was relatively small and limited to patients with end-stage renal disease, not the patients with CKD, who are more common in the clinical setting. Zhang et al [35] reported a predictive model for major adverse cardiovascular events prediction in patients with CKD undergoing percutaneous coronary intervention. They incorporated selected risk factors into a nomogram, while the discrimination needed to be improved (AUC=0.612). Current American College of Cardiology/AHA guidelines recommend MARCE as a novel clinical end point for patients with cardiovascular disease and CKD, for more accurate risk stratification and providing new pathophysiological insights. Thus, a predictive model aiming at the MARCE is warranted, especially for patients with AMI and CKD.

ML is an important supporting technology for AI that allows computers to learn, analyze, and construct models from input data, and then output predictions for new samples. Combined with electronic health record systems, ML could help doctors predict risk in cardiovascular disease, therefore aiding in clinical decision-making for patients. Khera et al [36] developed an XGBoost model, a neural network, and a meta-classifier model with patients from the American College of Cardiology Chest Pain-MI Registry and found that the XGBoost algorithm improved prediction performance for high-risk individuals. Advanced ML methods were also used to improve the prediction of cardiac risk based on radiomic profiling of coronary artery perivascular adipose tissue. However, the computationally heavy, long analysis times required with the model limited the application, and the model needed to better characterize the association of perivascular adipose tissue with the coronary atherosclerosis phenotype [37]. These studies indicated that ML is a reliable, novel method for clinical practice.

The SHAP analysis provided information complementary to traditional regression-based interpretation. Whereas LR and nomogram models summarize average population-level associations, SHAP decomposes each individual XGBoost prediction into feature-specific contributions. Thus, even with a small and clinically intuitive predictor set, SHAP may help clinicians identify whether a high predicted MARCE risk is mainly driven by hemodynamic severity, impaired renal function, elevated lactate, or medication-related factors. However, SHAP values should not be interpreted as causal effects, particularly for medication-related predictors such as diuretics, beta-blockers, and ACEI/ARB/ARNI, which may reflect confounding by indication, treatment eligibility, contraindications, or disease severity. Additionally, these medication-related predictors may partly capture physician decision-making and local treatment practice rather than baseline patient risk alone. Therefore, although they may improve risk prediction within the present dataset, their contribution should be interpreted cautiously when considering model transportability and implementation in health care systems with different treatment strategies.

In this study, we integrated ML workflows with MICE imputation, LASSO feature selection, cross-validation, hyperparameter tuning, and SHAP interpretation. These methods jointly supported the prediction of in-hospital MARCE in patients with AMI and CKD while retaining clinical interpretability.

Our study represents an application of established ML and nomogram modeling approaches to MARCE prediction in patients with AMI and CKD, rather than a fundamentally new modeling framework. Using 6 variables available early during hospitalization, clinicians may estimate MARCE risk and support early risk stratification. The nomogram showed higher discrimination than GRACE in this dataset; however, this comparison should be interpreted cautiously because both tools were evaluated in the same cohort, and the end point definitions differ from the original GRACE development setting. Therefore, the primary value of this study lies in its focus on a clinically high-risk cardiorenal population, the use of MARCE as a composite end point, and the integration of model interpretability with a parsimonious clinical prediction tool.

Although LASSO initially identified 7 predictors, the final 6-variable model was refined by excluding cardiogenic shock because of its clinical overlap with Killip class IV. This decision reflects a trade-off between statistical optimization and clinical interpretability. A fully data-driven model may retain all statistically selected variables, but redundant predictors can reduce model parsimony and complicate clinical interpretation. Therefore, we prioritized a parsimonious predictor set that preserved the main prognostic information while improving interpretability and usability in clinical settings. The sensitivity analysis using all 7 LASSO-selected predictors showed comparable performance, suggesting that this clinical refinement did not materially affect the main findings. Additionally, the extremely small learning rate selected during automated XGBoost tuning may represent a boundary solution of the optimization process rather than a biologically or clinically meaningful optimum. Although sensitivity analyses using conventional learning rates of 0.05 and 0.10 yielded similar AUC values, this finding highlights a limitation of automated hyperparameter optimization in relatively small clinical datasets and should be interpreted with caution.

Several limitations should be considered. First, despite external validation, the study sample was regionally limited, and the external cohort was modest in size. The external cohort included 157 patients and 35 MARCE events, which may affect the precision and stability of validation estimates, particularly for more complex ML models. Therefore, the external validation should be regarded as preliminary rather than definitive confirmation of model generalizability. Further multicenter studies with larger sample sizes are required to confirm model transportability, calibration stability, and clinical utility across different health care settings. Second, the study modeled the composite MARCE end point rather than individual end point components. Some components, such as recurrent MI and stroke, occurred infrequently, limiting stable component-specific prediction. Third, patients who died within 24 hours were excluded because complete predictor information within the predefined 24-hour assessment window was often unavailable, which may introduce survivorship bias and limit generalizability to the most critically ill patients. Fourth, because LASSO feature selection was performed once on the entire training set before cross-validation rather than nested within each resampling fold, cross-validation estimates in the training set may still be slightly optimistic. However, final model performance was evaluated in an untouched hold-out test set and an independent external validation cohort. Fifth, this retrospective study had moderate class imbalance, with a MARCE rate of approximately 25%. We did not apply oversampling, undersampling, or class weighting in the primary analysis to preserve the observed event prevalence and calibration of predicted risk. Therefore, threshold-dependent metrics should be interpreted cautiously. Finally, this study focused on in-hospital MARCE only; longer-term outcomes after discharge require future investigation.

### Conclusions

In this study, based on 6 important predictors, we developed 3 tree-based ML models and a nomogram for in-hospital MARCE risk prediction in patients with AMI and CKD. The models demonstrated clinically meaningful discrimination and may support early risk stratification, pending further multicenter prospective validation.

## Supplementary material

10.2196/96654 Multimedia Appendix 1 Patient characteristics, model performance, confusion matrices, pairwise area under the receiver operating characteristic curve comparisons, sensitivity analysis, and classifier hyperparameters.

10.2196/96654 Multimedia Appendix 2 Sensitivity analysis of extreme gradient boosting learning rate. Radar plot (left) and summary table (right) comparing the tuned model (learning rate=1.09×10^–^⁸) with fixed learning rates of 0.05 and 0.10 on the independent test set (n=161; threshold=0.50; major adverse renal and cardiovascular events as positive class).

10.2196/96654 Multimedia Appendix 3 Decision curve analysis of the nomogram and the Global Registry of Acute Coronary Events score.

10.2196/96654 Multimedia Appendix 4 Performance evaluation of the nomogram in the validation set: receiver operating characteristic curve (A), 400-resample bootstrapped calibration plot (B), decision curve analysis (C), and clinical impact curve (D).

10.2196/96654 Multimedia Appendix 5 Web-based dynamic nomogram for estimating the probability of in-hospital major adverse renal and cardiovascular events.

10.2196/96654 Checklist 1 TRIPOD checklist.

## Abbreviations

ACEI: angiotensin-converting enzyme inhibitor
AHA: American Heart Association
AMI: acute myocardial infarction
ARB: angiotensin II receptor blocker
ARNI: angiotensin receptor-neprilysin inhibitor
AUC: area under the receiver operating characteristic curve
CKD: chronic kidney disease
CKD-EPI: Chronic Kidney Disease Epidemiology Collaboration
DCA: decision curve analysis
DT: decision tree
eGFR: estimated glomerular filtration rate
GRACE: Global Registry of Acute Coronary Events
*ICD-10*: *International Statistical Classification of Diseases, Tenth Revision*
LASSO: least absolute shrinkage and selection operator
LR: logistic regression
MARCE: major adverse renal and cardiovascular events
MI: myocardial infarction
MICE: Multivariate Imputation by Chained Equations
ML: machine learning
RF: random forest
SHAP: Shapley additive explanations
TCM: traditional Chinese medicine
TRIPOD: Transparent Reporting of a Multivariable Prediction Model for Individual Prognosis or Diagnosis
XGBoost: extreme gradient boosting
