# Supplementary material for: Explainable Machine Learning for Predicting Major Adverse Renal and Cardiovascular Events in Acute Myocardial Infarction With Chronic Kidney Disease: Algorithm Development and Validation
Source: JMIR Med Inform. 2026 Oct 23;14:e96654. doi: 10.2196/96654 (PMC13717327; doi:10.2196/96654)
Supplement: Supplementary file 1 [file medinform-v14-e96654-s001.docx]

**Table S1. Clinical characteristics of patients who did or did not develop MARCE in the external validation set.**

| **Variables** | **No MARCE (n=122)** | **MARCE (n=35)** | **P value** |
| --- | --- | --- | --- |
| **Demographic** |  |  |  |
| Age(years) | 73.78±10.87 | 72.57±12.66 | 0.578 |
| Sex |  |  | 0.374 |
| female | 23 (18.85%) | 9 (25.71%) |  |
| male | 99 (81.15%) | 26 (74.29%) |  |
| **presentation characteristics** |  |  |  |
| Killip.class |  |  | <0.001 |
| 1 | 47 (38.52%) | 4 (11.43%) |  |
| 2 | 41 (33.61%) | 4 (11.43%) |  |
| 3 | 20 (16.39%) | 15 (42.86%) |  |
| 4 | 14 (11.48%) | 12 (34.29%) |  |
| **Medication at admission（%）** |  |  |  |
| Beta blockers |  |  | <0.001 |
| 0 | 32 (26.23%) | 22 (62.86%) |  |
| 1 | 90 (73.77%) | 13 (37.14%) |  |
| ACEI/ARB/ARNI |  |  | <0.001 |
| 0 | 59 (48.36%) | 29 (82.86%) |  |
| 1 | 63 (51.64%) | 6 (17.14%) |  |
| Diuretics |  |  | 0.755 |
| 0 | 39 (31.97%) | 13 (37.14%) |  |
| 1 | 83 (68.03%) | 22 (62.86%) |  |
| **Laboratory data** |  |  |  |
| Lactate(mmol/L) | 1.6 (1.3,2.2) | 2.3 (1.7,3.35) | <0.001 |
| BNP(pg/ml) | 853.5 (199.75,2663.25) | 1702.95 (581.5,4641) | 0.03 |
| Creatinine(μmol/L) | 142 (122.25,193.25) | 310 (166.5,504.5) | <0.001 |
| eGFR(ml/min/1.73 m^2^ ) | 40.1 (23.21,52.3) | 17.51 (10,30.91) | <0.001 |
| FBG(mmol/L) | 7.67±3.72 | 9.98±5.86 | 0.033 |
| D.Dimer(mg/L) | 0.62 (0.4,1.19) | 0.85 (0.58,1.35) | 0.044 |
| LVEF(%) | 53.45±12.63 | 43.75±12.59 | <0.001 |

Continuous variables are presented as mean (standard deviation) for normally distributed variables or median (interquartile range [IQR]) for non-normally distributed variables, while categorical variables are presented as number (percentage).

**Abbreviation: ACEI- angiotensin-converting enzyme inhibitor; ARB- angiotensin II receptor blocker; ARNI- angiotensin receptor neprilysin inhibitor; BNP- brain natriuretic peptide; eGFR- estimated glomerular filtration rate; FBG- fasting blood glucose; LVEF- left ventricular ejection fraction; MARCE- major adverse renal and cardiovascular events**

**Table S2. Ten-fold cross-validation results of AUC and accuracy of models in the train set**

| **Classifiers** | **AUC** | **Accuracy** |
| --- | --- | --- |
| Logistic | | |
| Fold-1 | 0.830 | 0.895 |
| Fold-2 | 0.832 | 0.737 |
| Fold-3 | 0.798 | 0.763 |
| Fold-4 | 0.866 | 0.921 |
| Fold-5 | 0.718 | 0.763 |
| Fold-6 | 0.981 | 0.946 |
| Fold-7 | 0.731 | 0.730 |
| Fold-8 | 0.981 | 0.919 |
| Fold-9 | 0.950 | 0.973 |
| Fold-10 | 0.849 | 0.757 |
| Average | 0.854 | 0.840 |
| XGBoost | | |
| Fold-1 | 0.910 | 0.795 |
| Fold-2 | 0.767 | 0.795 |
| Fold-3 | 0.832 | 0.789 |
| Fold-4 | 0.887 | 0.784 |
| Fold-5 | 0.762 | 0.811 |
| Fold-6 | 0.817 | 0.784 |
| Fold-7 | 0.954 | 0.757 |
| Fold-8 | 0.875 | 0.811 |
| Fold-9 | 0.917 | 0.811 |
| Fold-10 | 0.774 | 0.811 |
| Average | 0.850 | 0.795 |
| Random forest | | |
| Fold-1 | 0.905 | 0.895 |
| Fold-2 | 0.885 | 0.842 |
| Fold-3 | 0.778 | 0.789 |
| Fold-4 | 0.949 | 0.895 |
| Fold-5 | 0.693 | 0.868 |
| Fold-6 | 0.864 | 0.865 |
| Fold-7 | 0.944 | 0.919 |
| Fold-8 | 0.915 | 0.784 |
| Fold-9 | 0.772 | 0.865 |
| Fold-10 | 0.813 | 0.838 |
| Average | 0.852 | 0.856 |
| Decision tree |  |  |
| Fold-1 | 0.850 | 0.816 |
| Fold-2 | 0.891 | 0.842 |
| Fold-3 | 0.490 | 0.737 |
| Fold-4 | 0.776 | 0.842 |
| Fold-5 | 0.458 | 0.763 |
| Fold-6 | 0.760 | 0.865 |
| Fold-7 | 0.861 | 0.892 |
| Fold-8 | 0.880 | 0.811 |
| Fold-9 | 0.739 | 0.865 |
| Fold-10 | 0.754 | 0.784 |
| Average | 0.746 | 0.822 |

AUC, area under the receiver operating characteristic curve

**Table S3. Confusion matrices of four classifiers in the test set.**

| **Model** | **TP** | **FP** | **TN** | **FN** |
| --- | --- | --- | --- | --- |
| Logistic regression | 26 | 4 | 117 | 14 |
| XGBoost | 10 | 1 | 120 | 30 |
| Random forest | 27 | 10 | 111 | 13 |
| Decision tree | 26 | 8 | 113 | 14 |

**Abbreviations: TP, true positive; FP, false positive; TN, true negative; FN, false negative. MARCE was defined as the positive class.**

**Table S4. Pairwise AUC comparisons using the DeLong test.**

| **Comparison** | **AUC difference** | **P value** | **Interpretation** |
| --- | --- | --- | --- |
| XGBoost vs LR | 0.007 | 0.891 | Not significant |
| XGBoost vs RF | 0.012 | 0.806 | Not significant |
| LR vs RF | 0.005 | 0.924 | Not significant |
| XGBoost vs DT | 0.068 | 0.208 | Not significant |
| LR vs DT | 0.061 | 0.287 | Not significant |
| RF vs DT | 0.056 | 0.312 | Not significant |
| Nomogram vs GRACE | 0.110 | 0.033 | Significant |

AUC comparisons were performed using the DeLong test.

**Table S5. Sensitivity analysis of classifiers' predictive performance in the test set using seven LASSO-selected variables**

| **Index** | **Logistic** | **XGBoost** | **Random forest** | **Decision tree** |
| --- | --- | --- | --- | --- |
| AUC (95%CI) | 0.876 (0.799-0.951) | 0.886 (0.820-0.951) | 0.873 (0.803-0.943) | 0.821 (0.728-0.891) |
| Brier score (95%CI) | 0.092 (0.058-0.127) | 0.097 (0.063-0.131) | 0.108 (0.073-0.143) | 0.112 (0.075-0.149) |
| Accuracy | 0.882 | 0.814 | 0.851 | 0.870 |
| MCC | 0.667 | 0.434 | 0.588 | 0.631 |
| F1 score | 0.732 | 0.464 | 0.684 | 0.704 |
| Kappa | 0.658 | 0.376 | 0.587 | 0.622 |
| PPV | 0.839 | 0.812 | 0.722 | 0.806 |
| NPV | 0.892 | 0.814 | 0.888 | 0.885 |
| Sensitivity | 0.650 | 0.325 | 0.650 | 0.625 |
| Specificity | 0.959 | 0.975 | 0.917 | 0.950 |

AUC, area under receiver operating characteristic curve; MCC, Matthews correlation coefficient; PPV, positive predictive value; NPV, negative predictive value. Classification metrics were calculated with MARCE as the positive class at a fixed threshold of 0.50.

**Table S6. Confusion matrices of seven-variable sensitivity models in the test set**

| **Model** | **TP** | **FP** | **TN** | **FN** |
| --- | --- | --- | --- | --- |
| Logistic regression | 26 | 5 | 116 | 14 |
| XGBoost | 13 | 3 | 118 | 27 |
| Random forest | 26 | 10 | 111 | 14 |
| Decision tree | 25 | 6 | 115 | 15 |

TP, true positive; FP, false positive; TN, true negative; FN, false negative. The assumed hold-out test set included 40 MARCE-positive and 121 MARCE-negative patients.

**Table S7. Final hyperparameters of classifiers.**

| **Model** | **Hyperparameters** | **Final values** |
| --- | --- | --- |
| Logistic regression | Penalty / solver | No penalization / binomial logistic model |
| XGBoost | mtry; trees; min_n; tree_depth; learn_rate; loss_reduction; sample_size | 1; 150; 11; 4; 1.09014647938676e-08; 1.34974288892155e-07; 0.82437728113262 |
| Random forest | mtry; trees; min_n | 2; 500; 10 |
| Decision tree | tree_depth; min_n; cost_complexity | 5; 20; 0.001 |
